# Effects of removable clear dental aligners on the composition of the oral microbiome

**DOI:** 10.1080/20002297.2025.2579836

**Published:** 2025-11-17

**Authors:** Sara Peregrina, Andrea Peiró Aubalat, Avié Manohar, Alicia Benavente, Martha Torres-Carvajal, Toni Gabaldón

**Affiliations:** a Barcelona Supercomputing Centre (BSC-CNS), Plaça Eusebi Güell, Barcelona, Spain; b Institute for Research in Biomedicine (IRB Barcelona), The Barcelona Institute of Science and Technology, Baldiri Reixac, Barcelona, Spain; c Department of Odontostomatology – Orthodontics, Faculty of Medicine and Health Sciences, University of Barcelona, Barcelona, Spain; d Catalan Institution for Research and Advanced Studies (ICREA), Barcelona, Spain; e CIBER de Enfermedades Infecciosas, Instituto de Salud Carlos III, Madrid, Spain

**Keywords:** Dysbiosis, 16S sequencing, aligners, oral microbiome, diversity, dental plaque

## Abstract

Proper tooth alignment is important for oral and periodontal health, allowing better hygiene and reducing plaque build-up. While traditional braces are effective, clear aligners offer an aesthetic advantage and are also thought to promote better oral hygiene. However, their specific impact on the oral microbiome is not yet fully understood. This longitudinal study used 16S amplicon sequencing to study the oral microbiome (from saliva, subgingival, and supragingival samples) of 11 patients undergoing clear aligner treatment. Samples were collected at three time points: before treatment and at 3 and 6 months during therapy. Our results revealed large differences between the microbiomes of different oral sites but no significant overall changes in the oral microbiome composition due to orthodontic treatment. While some species-specific changes were observed, their effect sizes were very small. Although these results should be confirmed in a larger and more diverse cohort, they suggest that the treatment had a small or negligible impact. Given the observed stability of the oral microbiome in all three studied niches throughout the treatment and the known benefits to oral hygiene, clear aligners may present a favorable therapeutic alternative compared to fixed appliances.

## Article highlights

• The study revealed large differences in the composition of the microbiome between the different oral locations (saliva, subgingival and supragingival), although treatment with clear aligners did not significantly alter this overall composition.
• The stability of the oral microbiome during treatment with clear aligners, together with their hygienic advantages, positions clear aligners as favorable treatment options over fixed appliances.

## Introduction

Orthodontic appliances offer significant aesthetic and functional advantages. By aligning teeth, braces enhance facial appearance. Functionally, they correct bite issues, which can prevent excessive tooth wear, jaw problems, and difficulties in chewing and speaking. Furthermore, they promote even distribution of bite pressure, thus lowering the risk of long-term dental damage [1–3]. Proper dental alignment is crucial for maintaining oral and periodontal health, as malocclusion complicates oral hygiene and promotes plaque accumulation. This increased plaque burden elevates the risk of periodontal diseases, including gingivitis and periodontitis, and contributes to a higher incidence of white spot lesions and dental caries [4].

Despite these clinical benefits, the aesthetic and comfort drawbacks associated with fixed orthodontic appliances are widely recognized. Consequently, recent years have witnessed the development of alternative treatments that offer improved comfort and aesthetic discretion, notably removable clear aligners [5]. Owing to the development of suitable thermoplastic materials and computer-aided design computer-aided manufacturing (CAD-CAM) technology, removable, transparent aligners have been perfected, facilitating their widespread adoption even in complicated cases [6]. Clear aligners are a better option, especially for patients at risk for oral diseases such as gingivitis, as they have been reported to be more beneficial to periodontal health than fixed appliances [7]. In addition, because they are completely removable, they make it easier to maintain better oral hygiene and therefore reduce plaque accumulation compared to fixed appliances [8,9].

Orthodontics, whether removable or fixed, can create conditions that can alter the composition and balance of microorganisms in the mouth [10], [11]. Previous studies [1], [5], [12], [13] have shown that any type of orthodontics can lead to a change in the oral microbiome, especially increased retention of supra- and subgingival bacterial plaque throughout the treatment period. However, fixed appliances often lead to an increase in periodontopathogenic bacteria and the risk of caries [14]. While the clinical benefits of clear aligners for oral hygiene and periodontal health are evident, a comprehensive understanding of their specific impact on the oral environment, particularly the salivary microbiome and the composition of supra- and subgingival plaque, remains crucial, as changes in the composition of the oral microbiota can affect oral and systemic health [15].

This study aims to investigate these aspects, providing further insights into the microbiological impacts of clear aligner use. For this purpose, we followed a cohort of 11 first users of clear aligners, taking longitudinal samples before treatment and three and six months during treatment. At each time point, samples from total saliva and from supra- and subgingival plates were obtained, and the microbiome of each site was assessed via 16S metabarcoding. Our results showed only minor changes in the composition of the oral microbiome during the 6-month treatment, suggesting that this type of orthodontic treatment maintains the balance of the oral microbiome.

## Materials and methods

### Study design and sample collection

The study design was observational, analytical, longitudinal follow-up, non-experimental, and prospective for patients exposed to clear aligners. The participants were regularly monitored before the placement of the aligners and during treatment at 3 and 6 months. Therefore, the total follow-up period was 6 months. A total of 11 subjects (10 women, 1 man) voluntarily enrolled in this study were treated with Invisalign® LD30 multilayer thermoplastic polyurethane aligners made from methylendiphenyl diisocyanate and 1,6-hexanediol for a period of 6 months. All the subjects were aged between 17 and 64 years, with a mean age of 38 years. The study was conducted at the Universitat de Barcelona Dental Hospital from January 2022 to July 2023. All the subjects wore their appliances throughout the study period on both arches; after recruitment, all the necessary information about the study characteristics was provided, and written informed consent was obtained. The study was approved by the Ethics and Clinical Research Committee of the Universitat de Barcelona Dental Hospital (protocol numbers: 36/2021 and 37/21). The following inclusion criteria were applied: patients over 17 years old, no fixed or removable prostheses, adequate oral hygiene with no signs of severe gingivitis, the presence of target teeth to be analyzed, and at least 24 teeth with moderate crowding (less than 6 mm of space per arch). The exclusion criteria included patients who had received antibiotic treatment in the month prior to the study, had a history of routine use of oral antiseptics, had established gingivitis or severe periodontal disease, lacked first molars or central incisors, were pregnant or lactating women, or had severe systemic diseases. Once the voluntary subjects who met the inclusion and exclusion criteria were selected, they were instructed not to use antiseptics during the month prior to the recording, not to have consumed food or beverages for 1 h before, and not to have brushed their teeth in the last 5−7 h [16–18] At the time of sample collection, several oral health parameters were measured (see below).

The selected target teeth, representative of the sample, were the central incisors, canines, first premolars, and first molars from quadrants 1 and 3. A total of 88 teeth were analyzed at each time point. Supragingival and subgingival plaque samples were collected 5–7 h after tooth brushing.

Supragingival plaque was collected by gently sliding Gracey curettes (Hu-Friedy) over the vestibular (buccal), lingual (palatal), and interproximal surfaces of each of the 88 teeth, ensuring no contact with the gingiva or other oral tissues. The samples were then transferred into sterile Eppendorf tubes containing reduced transport fluid (RTF) and stored at −80 °C.

Additionally, subgingival samples were collected from the same tooth surfaces after polishing the tooth with a sterile prophylaxis brush. Two sterile endodontic paper points (size 30, Dentsply) were inserted per target tooth (central incisors, canines, first premolars, and first molars from quadrants 1 and 3) for 30 s – one through the vestibular sulcus and the other through the lingual sulcus – avoiding contact with other oral tissues. A total of 16 paper points were collected, pooled, and placed into sterile Eppendorf tubes containing RTF to preserve viable microorganisms at room temperature. The tubes were then immediately transported to an ultracold freezer (−80 °C).

Non-stimulated saliva samples were collected by drooling 2 mL of saliva into the DNA/RNA Shield SafeCollect Saliva Collection Kit (Zymo Research, R1211-E).

### DNA extraction

The ZymoBIOMICS® Microbiome Standard (ref #D6306) was used as a positive control in all the procedures. For the sub- and supragingival microbiome, DNA extraction was performed with the ZymoBIOMICS™ DNAMiniprep Kit (ref #D4300 ZymoResearch) as indicated by the manufacturer, except for a different preprocessing step, which was a necessary adjustment for efficient DNA extraction. This preprocessing included first thawing the samples on ice and then mixing them with vortexing for 5 min, after which the solution was transferred to a fresh 2 mL tube. The samples were subsequently centrifuged for 30 minutes at 16000 × g at 4 °C. The supernatant was removed, the pellet was gently resuspended in 200  μl of Milli-Q water, and then 550  μl of DNA/RNA shield (Zymoresearch) was subsequently added. Cell lysis was performed by securing the tubes in a bead beater fitted with a 2 ml tube holder assembly using the FastPrep-24 (MP Biomedical) instrument for 5 series of 1 min beating (5 min in total) with 5 min rest in between at maximum speed (6.5 M/S). Following this preprocessing, the protocol from the ZymoBIOMICS™ DNAMiniprep Kit was followed from step 3 onwards. DNA from saliva samples was extracted using the Quick-DNA Fungal/Bacterial Miniprep Kit (ZymoResearch) following the manufacturer's instructions.

## 16S amplification and sequencing

The V3–V4 region of the bacterial 16S ribosomal RNA gene was amplified using the following universal primers in a limited cycle PCR: V3–V4-Forward (5′-TCGTCGGCAGCGTCAGATGTGTATAAGAGACAGCCTACGGGNGGCWGCAG-3′) and V3–V4-Reverse (5′-GTCTCGTGGGCTCGGAGATGTGTATAAGAGACAGGACTACHVGGGTATCTAATCC-3′). Then, full-length Nextera adapters with barcodes for multiplex sequencing were added in a second PCR step, resulting in the sequencing of ready libraries. Sequencing was performed via Illumina MiSeq with 2 × 300 bp reads using v3 chemistry at the Genomics facility of the Centre for Genomic Regulation (CRG, Barcelona). Two bacterial mock communities from the BEI Resources of the Human Microbiome Project (HM-276D and HM-277D) were amplified and sequenced in the same manner as all other samples. Negative controls for PCR amplification were also included in parallel, using the same conditions and reagents.

## 16S amplicon sequence data analysis

Raw V3‒V4 16S amplicon sequencing data (PRJNA1284261) was provided by the sequencing facility in fastq format without barcodes and adapter sequences. Sequencing reads were analyzed with the Dada2 pipeline v1.30.0 [19]. First, we visualized the quality profiles using the plotQualityProfile function to determine the filtering parameters. Low-quality reads were filtered out by using filterAndTrim, and error models were learned from the data. Data obtained from subgingival plaques were trimmed at 290 for forward (F) reads and at 255 for reverse (R) reads. Supragingival plaque reads were trimmed at F 290 and R 240, and saliva samples were trimmed at F 285 and R 230. For all the samples, we set the trimLeft parameter to 10. F and R reads were merged using the mergePairs function and subsequently used to construct the amplicon sequence variant (ASV) table. Chimeric ASVs were removed with removeBimeraDenovo. Taxonomy was assigned by mapping to the SILVA database (v138) [20].

We used the phyloseq R package v1.46.0 [21] in subsequent analyses. We first integrated the ASV and taxonomy tables with their corresponding metadata into a phyloseq object. All negative control samples were removed before analysis (they produced fewer than 55 reads after filtering and trimming, indicating negligible contamination with reagents). Sequences assigned to mitochondria or chloroplasts were removed. A filter to remove samples with fewer than 1000 reads was applied, but all samples passed the filter.

We removed low-abundance taxa, keeping taxa that had more than 50 reads in more than 4 samples in this study; except in the case of the subgingival microbiome, samples with more than 50 reads in more than 3 samples were considered. The *estimate_richness* function was used to compute different alpha diversity measures, including the observed richness, Shannon index and Simpson index. A Wilcoxon signed rank test was run with the function *wilcox.test* from *stats,* and the ggplot2 package v3.4.4 was used for visualization.

Owing to the compositional nature of these data [22], we used a centred-log ratio (CLR) transformation of the data in subsequent analyses. We performed 0-replacement, using the CZM method in *cmultRepl* from *zCompositions* (v. 1.4.0.1), and CLR transformation, with *codaSeq.clr* from *CoDaSeq* (v. 0.99.7).

For beta-diversity analyses, Aitchison distances were calculated with the function *aDist* from *robCompositions* (v. 2.4.1). Differences in the distance between samples grouped by each categorical variable were assessed with a permutational multivariate analysis of variance (PERMANOVA) test using *adonis2* from *vegan* (v. 2.6.4). For each taxonomic rank, *p*-values for the comparisons (one for each categorical variable) were corrected using Benjamini-Hochberg (BH) correction. Following this procedure, we examined the relationships between the variables and the overall microbial composition of the samples. Distances were plotted in multidimensional plots for some of the variables that were found to be significant by the adonis test using plot_ordination function from the phyloseq package (v. 1.46.0).

We next performed a differential abundance analysis using the CLR-transformed data for each taxonomic rank as the dependent variable. To test for differentially abundant taxa according to two periods of time spent wearing aligners (T0–T1 and T0–T2), mixed-effect linear models were applied with *lmer* s from *lmer4* (v. 1.1.35.1), separately for each group. The sex and age variables were passed as fixed effects to correct for the likely differences caused by these variables. The subject variable was passed as a random effect to adjust for individual differences. An ANOVA test was run on the mixed-effect models with the function *Anova* from *car* (v. 3.1.2) to obtain *p*-values for the effect of each variable. *p*-Values for the effect of the Stage variable were corrected using BH correction.

### Clinical oral health parameters measurements and analytical procedures

At the time of sample collection, several clinical parameters of oral health were assessed for each patient: the plaque index (IP), gingival index (ISG), probing depth (PS), bleeding on probing (SS), DMFT calculation (referred to as CAOD in Spanish) calculation (CAOD), and salivary pH. These parameters were measured before starting treatment, at 3 months and at 6 months.

To assess the **plaque index**, the thickness of the supragingival plaque deposited on the gingival margin surface was measured with the tip of a periodontal probe (Hu-Friedy, Chicago, IL, USA, gray), and four surfaces were examined: vestibular, lingual, mesial and distal (interproximal points). The extent of gingival inflammation was evaluated using the **ISG** as described by Löe and Silness (1963). This index assesses changes in gingival color and texture prior to the onset of bleeding and provides standardized criteria for scoring gingival inflammation. Four surfaces of each selected representative tooth (buccal, lingual/palatal, mesial, and distal) were examined, with each surface assigned a score ranging from 0 to 3. The **probing depth** index was used to calculate the distance from the gingival margin to the bottom of the periodontal sulcus (junctional epithelium). It was measured using a millimeter periodontal probe (Silver CP12 Hu-Friedy) at six points on each target tooth, including the mesial, middle, and distal aspects in both the buccal and palatal/lingual regions. **Bleeding on probing** was assessed at each point using Lindhe's dichotomous index (1983), which involves dividing the number of sites with bleeding by the total number of sites explored. For the evaluation of the **DMFT (CAOD) index**, the following diagnostic criteria were established: the presence of dental caries, missing teeth, and dental restorations. These conditions were recorded at the level of the tooth surface using the following standardized abbreviations: D (decayed): the number of teeth with untreated caries; M (missing): the number of teeth lost due to caries; F (filled): the number of teeth restored (filled) because of caries; and T (tooth): the count per tooth. The sum of these values was then divided by the total number of teeth present in the individual and expressed as a percentage. **Salivary pH** was determined using universal test strips (Universal Cexun) to measure the acidity or alkalinity of saliva.

For each parameter, the Shapiro–Wilk test was first applied to assess the normality of the data distribution. Parameters that met the assumption of normality (PS, IP) were analyzed using a paired t-test, whereas parameters that did not follow a normal distribution (ISG, SS, CAOD, pH) were analyzed using the paired Wilcoxon signed-rank test. In addition, Spearman's rank correlation coefficient was calculated to assess the relationships between diversity metrics and the different clinical oral health parameters.

## Results

### Assessing oral microbiome composition in saliva and supra- and subgingival plaque

**Figure 1.**
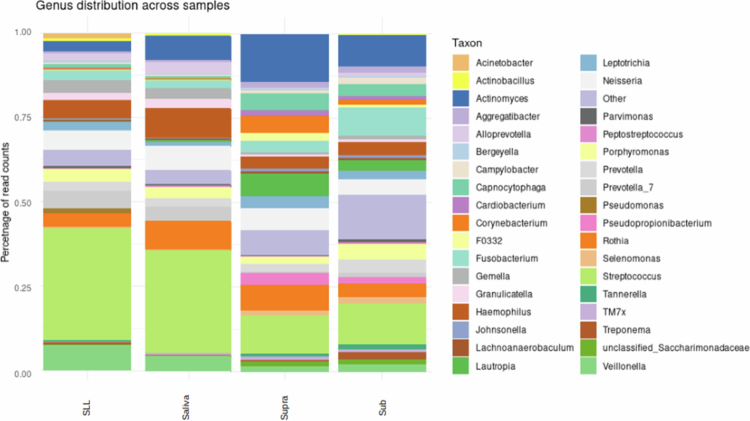
Relative abundance of the 35 most abundant genera across different sample types (saliva, supragingival (Supra), and subgingival (Sub)) obtained in this study, and comparison to a control cohort from a previous study of saliva from healthy subjects (SLL) [23]. Genera beyond the 35 most abundant genera are agglomerated in the ‘other’ category.

To assess potential changes in the oral microbiota resulting from the use of invisible dental aligners, we collected samples from saliva, as well as from supra- and subgingival plaques, from eleven volunteers (10 females, 1 male, aged 17−64 years) at three time points: just before the start of orthodontic treatment (T0) and at 3 (T1) and 6 (T2) months after the start of the treatment. During these visits, several oral health parameters were evaluated and recorded (see Materials and Methods, Table S1). The samples were collected (see Materials and Methods), frozen and stored until subsequent analysis. Following DNA extraction, we amplified the V3‒V4 regions of the 16S rRNA gene and sequenced the amplicons using Illumina MiSeq technology (see Materials and Methods). Sequencing libraries and a sufficient number of reads for further analysis was obtained for 33 saliva samples (average of 61,1165 reads per sample), 33 supragingival plaque samples (72,463), and 29 subgingival plaque samples (75,324). After quality clipping, we computationally assigned taxonomy to the amplicons (see Materials and Methods). Our analysis revealed typical oral microbiota compositions at the genus level (Figure 1), with *Streptococcus* being the most abundant genus in all sample types. Notably, the saliva from our cohort had an overall bacterial composition similar to the salival microbiome of a previously analyzed cohort of healthy persons in Spain [23]. Supra- and subgingival plaques clearly had compositions that were distinct from those of saliva samples, with relatively fewer *Streptococcus* and more *Lautropia* and *Leptotrichia*, among other differences (Figure 1). The relative taxa abundance at the genus level in each sample is shown in Supplementary Figure S1.

**Figure 2.**
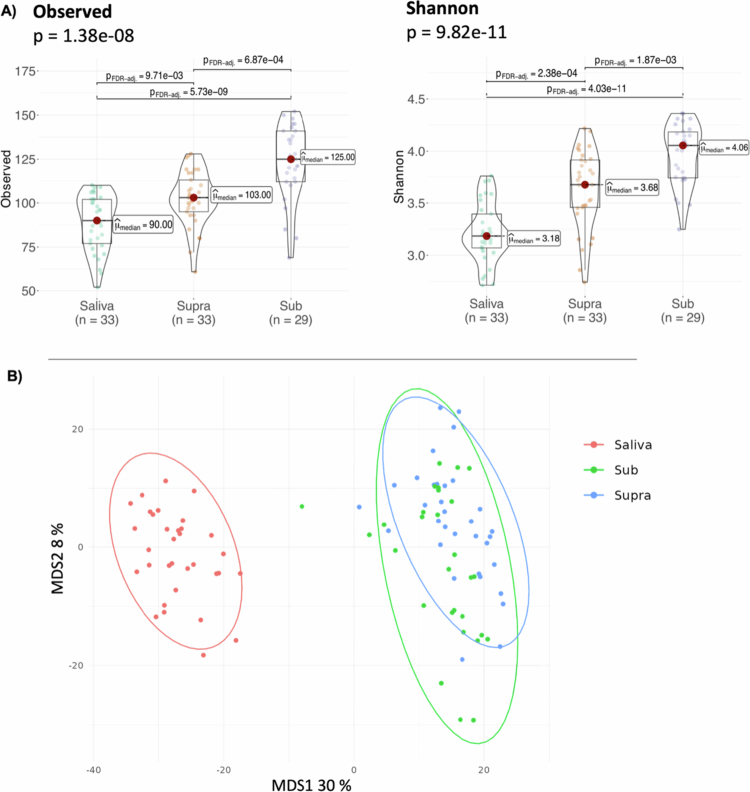
Overall composition comparing the different types of microbiome: saliva, sub- and supragingival plaque samples. (A) Alpha diversity and observed and Shannon indices. (B) Beta diversity (MDS).

Differences between the microbiomes of samples from the three oral sites were also very apparent when assessing overall alpha (within sample) and beta (between samples) diversity (Figure 2). Two alpha diversity differences across sites were statistically significant for both the observed and Shannon indices, which measure species diversity and evenness within each sample (see Materials and Methods, Figure 2A). Saliva samples had less diverse microbiomes, followed by supragingival and subgingival communities, in that order. Differences between samples, as represented using the Aitchison distance and Multidimensionality Scaling (MDS) plots, depict saliva as the most distinct sample type, with supra- and subgingival samples overlapping partially, with interindividual differences much greater in subgingival samples as compared to the two oral sites.

**Figure 3.**
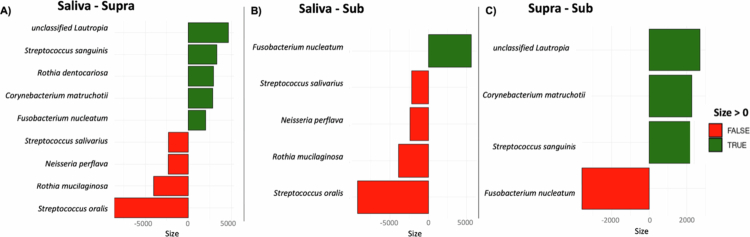
Differential abundance of taxa at the species level comparing the different types of microbiomes: saliva, sub- and supragingival plaque samples. (A) Saliva vs. supragingival plaque samples. (B) Saliva vs. subgingival plaque samples. (C) Supra vs subgingival plaque samples.

To assess the nature of these variations in more detail, we used linear models to establish statistically significant differences in the abundance of bacterial species. The results are consistent with massive differences in species abundance and include hundreds of differentially abundant species with very large effect sizes (a measure that quantifies the degree of difference in relative abundance) (Supplementary Table S1). Figure 3 represents the differentially abundant species with an effect size greater than 2000. Consistent with the overall composition results for each type of microbiome, more differences were found in the saliva sample group. Saliva samples had significantly more *Streptococcus oralis*, *Rothia mucilaginosa*, *Neisseria perflava*, and *Streptococcus salivarius*. In the supragingival plaque microbiome, compared to saliva and subgingival plaque, the following taxa are increased: *Corynebacterium matruchotii*, *Streptococcus sanguinis* and *unclassified Lautropia*. In addition, in supragingival plaque, we detected an increase in the taxon *Rothia dentocariosa*, which affects only saliva. Finally, the taxon *Fusobacterium nucleatum* is found to be more abundant in subgingival plaque than in supragingival plaque and more abundant in supragingival plaque than in saliva (Figure 3).

### High stability of oral microbiome during the orthodontic treatment

**Figure 4.**
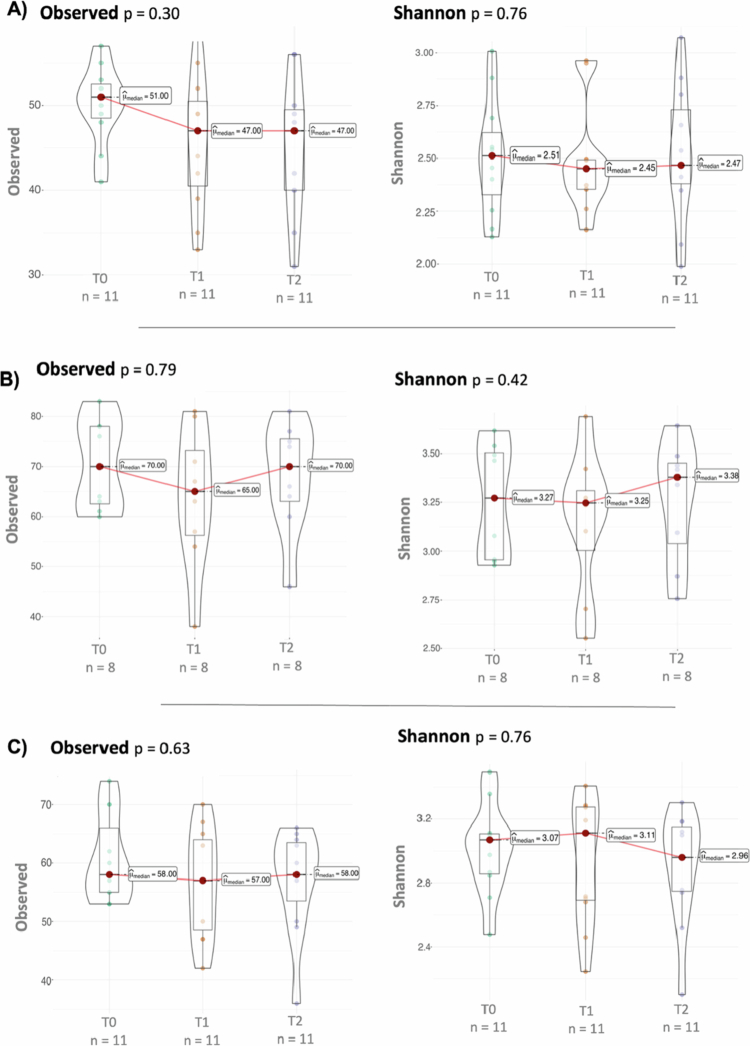
Box plots comparing two alpha diversity measures across the different treatment time points (T0, T1 and T2) in samples from (A) saliva, (B) subgingival plaque, and (C) supragingival plaque.

We next compared samples across the treatment period for each site, considering their paired nature (e.g. samples were taken from the same individuals at each time point). We first assessed changes in alpha diversity using the observed and Shannon indices. Our results revealed no significant differences in any diversity index when comparing paired samples before or after intervention, after 3 or after 6 months, in any of the three types of microbiomes studied in this study (Figure 4).

**Figure 5.**
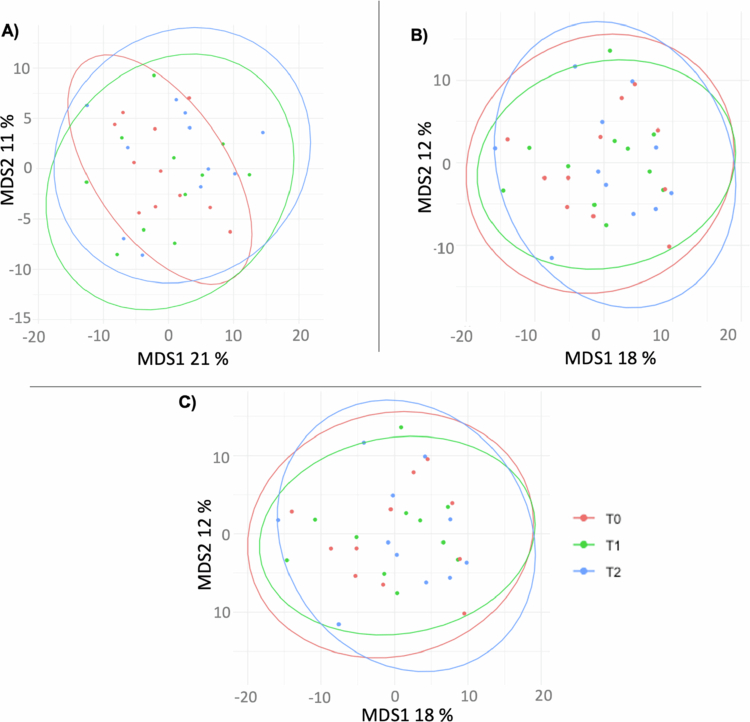
MDS plots using Aitchison distance between the microbial composition at the genus level of samples from saliva (A), subgingival plaque (B) and supragingival plaque (C). Each sample is represented by a dot colored according to the collection time.

We next performed beta diversity analysis to compare community structures between pre- and postintervention samples at the three oral sites. We observed no clear clustering of samples depending on the collection time, as shown in a multidimensional scaling plot based on overall microbial composition and Aitchison distance (Figure 5). Similarly, we did not detect statistically significant differences for samples from the same site at different time points, as assessed with the Adonis test.

We then performed a statistical analysis to detect significantly differentially abundant taxa at the species and genus levels according to the treatment time. Specifically, we evaluated the effect of the treatment time (T1, T2) by comparing it with the T0 baseline for each oral site separately, using an effect size threshold of 0.693, corresponding to a fold change of 2. In contrast to the previous across-site comparisons, these analyses revealed very few differentially abundant taxa and very small effect sizes, rarely exceeding an effect size of 2 (Supplementary Table S2).

**Figure 6.**
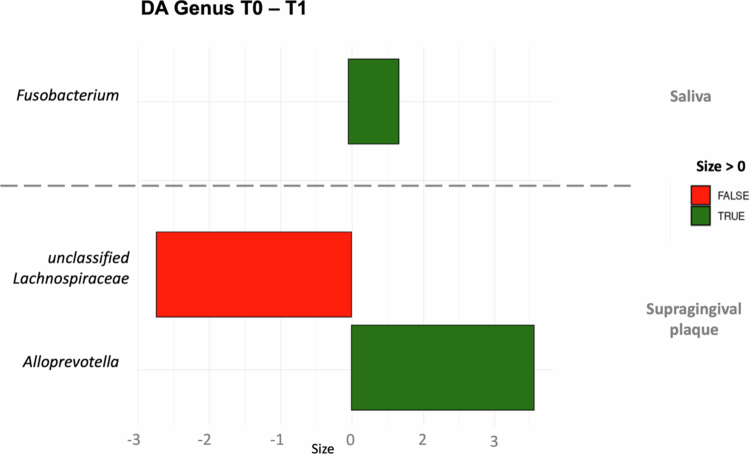
Differential abundance taxa comparing T0 vs. T1 in salivary microbiome samples and supragingival plaque microbiomes at the genus level.

We performed all comparisons between groups in the different microbiome types and found only 3 taxa that were differentially abundant at the genus level whose abundance varied significantly after 3 months. One taxon in the salivary microbiome and 2 taxa in the supragingival plaque microbiome. No taxa were reported as differentially abundant in the subgingival microbiome samples. A positive size indicates that this taxon is elevated in the group to which T0 is compared, i.e. T1 or T2 (Figure 6).

However, given the limited sample size of our study, more subtle changes may have not reached statistical significance, and we consider it interesting to report some of the observed trends below the significance threshold. These include changes in abundance in the salival microbiome, involving 11 taxa at the species level and 5 at the genus level after 3 months and 8 species and 6 genera after 6 months (see Supplementary Table S3 and Figure S2).

We also carried out comparisons between different clinical oral health parameters: plaque index, gingival index, probing depth, bleeding on probing, CAOD calculation, and salivary pH. No differences were reported in any of these indices when comparing each at 0 and 6 months (*p* > 0.05). However, statistically significant differences were found in the PS and SS at 3 months compared to baseline (T1–T0). Likewise, the differences were also significant when 3 and 6 months (T2–T1), were compared, with an improvement in the ISG observed at the third assessment. Correlations were also made between different metrics of microbial diversity and clinical parameters. Only the correlation between the Shannon diversity index of the supragingival microbiome and the plaque index was found to be significant (R = 0.344, *p* = 0.049).

## Discussion

The maintenance of a balanced oral microbiome is important, as alterations in its composition can predispose individuals to a range of oral health issues, including white spot lesions, dental caries, or gingivitis [24]. Furthermore, given the high connectivity of the oral cavity with the vascular, respiratory and digestive systems, changes in the oral microbiome can also affect systemic health [15]. It is well documented that orthodontic appliances can influence the composition of the oral microbiome [8], [24]. However, given their more recent development, there is a scarcity of studies analyzing the impact of clear removable aligners on the oral microbiota [1,25–28].

Here, we performed a longitudinal investigation on the impact of clear aligner therapy on the oral microbiome across distinct oral sites (saliva, subgingival plaque, and supragingival plaque) in a cohort of 11 patients over a six-month period. Our findings indicate very large differences in microbial composition across various oral sites, an observation that is consistent with the well-established ecological niches within the oral cavity [29–32]. Comparatively, we found only minor differences when comparing the oral microbiome of the same oral site across the treatment period.

For the differences between different oral sites, our results agree with previous assessments [33] that alpha diversity increases as the medium becomes more anaerobic, from saliva, supragingival plaque and subgingival plaque. Multiple studies [34], [35] have demonstrated significant differences in bacterial composition between saliva and dental plaque, encompassing both supragingival and subgingival microbiota. Consistent with these findings, our study revealed distinct microbial community structures among subgingival plaque, supragingival plaque, and saliva.

In addition, previous studies [36] revealed an increase in the abundance of the salival microbiome of anaerobic species typical of the subgingival niche and poorer oral and periodontal health [35], [37]. In this regard, the observed increase in the abundance of *Fusobacterium* in saliva during the treatment with aligners may be relevant, although the effect size was very small. Our results detect only very minor changes in the oral microbiome at all three oral sites that can be attributed to orthodontic therapy. In addition, no significant differences were observed in any of the oral health parameters between baseline and six months (*p* > 0.05), suggesting stability in the clinical oral health status of the participants during the treatments.

Although changes in the relative abundances of different species are found at each site and individual for different time points, the general lack of consistency of these changes among different individuals suggest most changes are likely stochastic fluctuations unrelated to the treatment. In this context, the small effect size of the few significant changes detected suggests a very limited physiological impact or biological relevance. Admittedly, the limited size and heterogeneous nature of our cohort may hinder the detection of statistically significant changes. Further studies with larger cohorts should corroborate our findings before our conclusions can be generalized. However, clear differences with sufficient statistical support were found across sites, suggesting that changes due to treatment are comparatively much smaller. Nevertheless, some statistically-significant changes were detected during the treatment for saliva and supra-gingival plaque, albeit with very small effect sizes. Among the observed trends, some affect species that have been previously associated with different oral health conditions. For instance, in saliva, we detected a decreasing trend in the species *Corynebacterium matruchotii*, which was consistent after 3 and 6 months of treatment. This species has been found to be related to parameters of good oral health [38], [27]. Conversely, *Actinomyces viscosus* and *Actinomyces gerencseriae* were found to have an increasing trend in the salivary microbiome at 3 and 6 months, respectively, and this genus is associated with caries formation [39], [9]. Only minor changes, which were not consistent across time points, were observed in the supragingival plaque, while no significant change was detected in the subgingival plaque. However, previous studies have also shown that invisible orthodontic treatment can acidify the environment [40], [28] Furthermore, aligners promote the adhesion of different microbes, including pathogenic microbes and species of the genus *Candida*, as has been observed in previous studies [40], which, as eukaryotic species, is beyond the scope of this study.

Some previous studies have also found no significant differences in composition after clear aligner therapy in supra [9] and subgingival plaque [41]. However, other studies have reported a decrease in the species richness present in saliva samples [42] and supragingival plaque samples [12], [43]. Despite the fact that no significant results were found in our study, a small trend in the decrease in richness over time was visually apparent, although the limited sample size may have resulted in a lack of statistical power. Aligners create a closed environment that may inhibit the growth of some bacteria, and thus, a decrease in both richness and diversity may be encountered [27].

Similarly, studies have shown that periodontally healthy individuals exhibit greater diversity in bacterial populations [23]. Our results coincide with those of Zhao et al., who evaluated the effects of aligners on the bacterial community during a 6-month follow-up period and found no significant changes in overall biodiversity compared to baseline [44]. In contrast, Wang et al., in a sample of 5 patients treated with fixed orthodontics, 5 with aligners and 5 controls, reported a decrease in the diversity of the oral ecosystem as a result of treatment, regardless of the appliance used. It should be noted that the present study was conducted at a single time point and did not take into account interindividual differences or baseline time [25].

Therefore, our results are consistent with previous studies showing no or very small impact of clear aligners on the salival, supergingival or subgingival microbiota. This small impact of the microbiota may be related to the fact that, compared to fixed aligners, removable clear alignments promote and allow the maintenance of better oral hygiene during treatment, since it is important to maintain strict oral hygiene in all patients with any type of orthodontic treatment in order to have good oral health [40], [28]. Studies have also shown that, in contrast to those who use fixed orthodontic devices, patients who use removable orthodontic devices demonstrate improved periodontal health after treatment [28], [45], [46]. Therefore, beyond correcting malocclusion, this treatment also enhances overall oral health [47]. In addition, our findings support the idea that the materials used do not induce large changes in the composition of the oral microbiome. This study did not assess the potential formation of bacterial biofilms on the surface of the aligners. However, the observed stability of the oral microbiome during treatment suggests that if biofilms are formed, they do not seem to significantly impact the composition of the oral microbiota. Nevertheless, orthodontic therapies can continue to improve with the development of new antibacterial materials that do not allow the growth of certain bacteria related to poor oral health, as well as new cleaning methods that help improve the health of patients with this type of treatment [48].

With respect to the clinical parameter results, these findings could be associated with temporary gingival enlargement at 3 months of treatment, resulting from a change in the oral ecosystem due to the aligner factor. Since no differences were found when comparing baseline with 6 months (T2–T0), the results seem to indicate that the initial conditions were restored after 6 months of treatment. These findings are consistent with those of Azaripour et al., who found no differences in PS when comparing 6 months of treatment with baseline [49].

Miethke et al. evaluated the Silness and Loë gingival indices, probing depth, and gingival bleeding in patients treated with aligners versus those treated with fixed orthodontics at three appointments spaced at intervals of 3–4 weeks. As the authors point out, one limitation of the study was that it did not assess the baseline situation prior to the placement of the appliances, so it did not take into account interindividual variability, unlike our study. Notably, in their results, all indices showed improvement from the first to the third assessment, all of which occurred after appliance placement. These results coincide with our improvement observed at 6 months in probing depth and bleeding on probing compared to 3 months [50].

Our study has several limitations, including a reduced sample size and a large bias toward female participants. Although the use of a longitudinal sampling strategy alleviates the problem of interindividual variation, larger and more balanced cohorts should be used in further studies to confirm the low impact of aligners on the oral microbiota. Moreover, no control group not using clear aligners was included, preventing us from assessing the stability of the oral microbiome in untreated patients. Although the oral microbiome has been shown to have a higher stability than other sites do [51], such a comparison would be important for assessing whether that stability is affected by the use of dental aligners. Finally, given the study design, it cannot assess long-term changes after the study period (6 months) or changes in the metabolic potential or strain- or specific-specific levels not detectable with the level of resolution provided by the 16S V3-V4 amplicon marker.

## Conclusion

Previous studies [5], [12] have demonstrated that fixed orthodontics presents a higher risk of gingivitis and periodontitis due to the difficulty of proper cleaning around the brackets and wires. Invisible orthodontics, being removable, allows for better hygiene and, therefore, a lower incidence of periodontal problems. As a result of this study, invisible orthodontics is a better alternative, as demonstrated in this study, which hardly results in imbalances in the oral microbiome and is more comfortable and aesthetic. However, advances and improvements in cleaning and aligner materials can still be developed to inhibit the growth of some bacteria that have been linked to poor health and imbalance of the oral microbiome.

## Ethical approval

The study was approved by the Ethics and Clinical Research Committee of the Universitat de Barcelona Dental Hospital (protocols number: 36/2021 and 37/21).

## Supplementary Material

Supplementary Material TableS1

Supplementary Material TableS2

## Data Availability

Sequencing data obtained in this study are available in the short read archive, Bioproject PRJNA1284261.
